# Correction

**DOI:** 10.1111/cas.15669

**Published:** 2023-02-14

In an article^1^ titled “Tumor‐derived immunoglobulin like transcript 5 induces suppressive immunocyte infiltration in colorectal cancer” by Shi, Wenjing; Zhang, Fang; chen, xiaozheng; Wang, Shuyun; Zhang, Haiqin; Yang, Zijiang; Wang, Guiying; Zheng, Yan; Han, Yali; Sun, Yuping; Gao, Aiqin, the following errors were published:

Under the Acknowledgements section, the foundation number of the Shandong Provincial Natural Science Foundation (ZR202103040716) should be ‘ZR2021MH268’.

The authors apologize for the error.
